# Functional Articulating Antibiotic Spacers for Chronic Native Septic Knee Arthritis

**DOI:** 10.1016/j.artd.2024.101329

**Published:** 2024-06-27

**Authors:** Levent A. Ozdemir, Andrew E. Apple, C. Lowry Barnes, Benjamin Stronach, Simon C. Mears, Jeffrey B. Stambough

**Affiliations:** a Department of Orthopaedic Surgery, University of Arkansas for Medical Sciences, Little Rock, AR, USA; b Department of Orthopaedic Surgery, Tulane University, New Orleans, LA, USA

**Keywords:** Articulating antibiotic knee spacer, Chronic septic knee arthritis, Metal-on-polyethylene knee spacer, Native knee septic arthritis

## Abstract

**Background:**

Semipermanent functional spacers are now utilized for prosthetic joint infection in an attempt to avoid another surgery with 2-stage treatment. This study evaluates the results of metal-on-polyethylene articulating spacers for the treatment of chronic native septic knee arthritis.

**Methods:**

This is a retrospective review of 18 patients treated with metal-on-polyethylene articulating antibiotic spacers constructed with all-polyethylene tibial components or with polyethylene inserts (PIs) with Steinmann pins or screws for chronic native knee infection. Demographic information, spacer construct type, prior knee surgery, complications, infecting organisms, infection eradication, and functional results were analyzed.

**Results:**

Of 18, 8 (44%) spacers were all-polyethylene tibial components and 10 (56%) were PI. Of 18 patients, 5 (28%) experienced spacer complications. Of 18 patients, 12 (67%) underwent a second reimplantation surgery (mean 106 days), while 6 (33%) retained their spacer (average duration 425 days). The PI group performed better in Knee Injury and Osteoarthritis Outcome score for Joint Replacement according to minimum clinically important difference and patient acceptable symptom state (PASS) criteria. The overall reimplantation group achieved Knee Injury and Osteoarthritis Outcome score for Joint Replacement PASS criteria and minimum clinically important difference criteria, while the maintained articulating spacer group did not achieve PASS criteria; however, they did reach minimum clinically important difference.

**Conclusions:**

Functional articulating spacers are a viable treatment for chronic, native knee septic arthritis. The PI patient group had a greater improvement in Knee Injury and Osteoarthritis Outcome score for Joint Replacement scores and had no significant difference in reimplantation rate as the all-polyethylene tibial components patient group. Both planned 2-stage reimplantation and longer-term spacer retention show promising results for this difficult clinical problem.

## Introduction

The knee is the most infected synovial joint, accounting for 40% to 50% of joint infections [1]. Initial treatment often includes open vs arthroscopic irrigation and debridement with tailored antibiotic therapy. If the knee has marked degenerative changes or chronic osteomyelitis, resection of damaged bone is necessary as irrigation and debridement, or antibiotics alone, are unlikely to resolve the infection [2]. Function is poor when the joint is arthritic and the bony resection may result in instability, worsening the degenerative changes [3]. In these cases, a 2-stage protocol, similar to that used for prosthetic joint infection, has proven to be successful in case series using static antibiotic cement spacers, antibiotic cement beads, or antibiotic cement molded spacers for the initial stage of treatment [[1], [2], [3], [4], [5], [6], [7], [8], [9], [10], [11], [12], [13]].

A potential one-and-a-half stage procedure for treating periprosthetic joint infection involves a functional articulating spacer featuring a metal femoral component, a tibial polyethylene insert, and high-dose antibiotic cement, with the prospect of longer-term spacer retention. Typically, these patients opt to keep their spacer in-situ and delay 2-stage reimplantation past 3-6 months [14]. This approach has advantages in both function and potential for implant retention, which may obviate a second-stage reimplantation or delay the second surgery if the need arises for future revision arises due to implant loosening or failure. A recent study with 3-year average follow-up demonstrated that these spacers continue to function well in terms of pain relief and joint range of motion [10]. The articulating spacer has particular advantages for the septic knee patient as other alternatives have relatively poor function and require a second stage. Patients with chronic knee infections often have comorbidities that make the patient a poor candidate for sequential surgeries [15]. This study evaluates the use of an articulating spacer constructed with metal-on-polyethylene (MoP) implants and high-dose antibiotic cement for treating previously infected native knees with resultant septic arthritis. The aim of this study is to compare outcomes in patients with all-polyethylene tibial components (APT) vs those with polyethylene inserts (PI). The basic difference of the constructs is as so: The APT spacer is made to function as its own as a polyethylene component and potentially has more rotational control because it has a keel built in for tibial bone engagement. The PI is a flat surface and does not have ridges for cement interdigitation, so it requires manual preparation and doctoring by the surgeon to roughen up, add a screw or Steinman pin and build up with cement to add some element of tibial engagement. There are lower costs associated with PI spacers vs the APT spacer without a compromise in function for patients with prosthetic joint infection (PJI) [16]. Based on our prior study we believe PI and APT spacers will perform similarly in terms of function and durability without differences in reinfection with the understanding likely implant cost savings using a PI insert.

We evaluated spacer retention, spacer-related complications, microorganism profiles, spacer cost, Knee Injury and Osteoarthritis Outcome score for Joint Replacement (KOOS JR) scores, and functional outcomes in the 2 cohorts. Our secondary objective it to assess differences in patient outcomes requiring a second stage surgery vs those who left their spacers in place.

## Material and methods

After institutional review board approval, a retrospective review was conducted to identify all patients who underwent placement of an articulating antibiotic knee spacer by 1 of 5 arthroplasty trained adult reconstructive surgeons at a single tertiary care academic center from September 2016 to August 2020. Inclusion criteria were age greater than 18 years and placement of an articulating antibiotic knee spacer for active, chronic, native knee infection. Native knees were defined as those that had not previously undergone total or partial knee arthroplasty. We included a total of 6 patients in the study who had prior arthroscopic incision and drainage or arthroscopic surgery (Table 1). Data from electronic medical record review were collected on patient demographic information, spacer constructs, history of prior knee surgeries, spacer-related complications, microorganism profiles, infection recurrence, spacer cost, spacer longevity, knee range of motion, reimplantation constructs, KOOS JR, and overall functional outcomes.

**Table 1:** Patient clinical data.

Patient	Age	Sex	BMI	Prior knee surgery/intervention	Spacer type	Culture results	Spacer-related complications	Spacer duration (d)	Recurrent infection	Replant implants	Overall function
1	67	M	31	ATS I&D	APT	MSSA		73	N	VVC	No gait aids, ROM 0-125
2	77	M	26.1	Steroid injection	APT	S. cohnii urealyticus		557	N	N/A	No gait aids, ROM 0-110
3	60	M	29.3	I&Dx2	APT	MSSA		174	N	VVC, T cone	No gait aids, ROM 0-125
4	50	M	44.8	11 previous for soft-tissue coverage	PI	Beta hemolytic strep		75	N	Hinge, T cone	No gait aids, ROM 0-120
5	57	M	44.3	External fixation, tibial plateau ORIF	PI	MSSE		168	N	Hinge, T cone	No gait aids, ROM 0-110
6	45	F	47.25	N/A	APT	Negative		75	N	VVC, T cone	L knee replant doing fine, ended up with PJI R knee, ROM 0-110
7	66	M	28.03	LCL recon, ATS, multiple I&Ds	PI	MRSA	Draining sinus, ROM 0-15	88	N	N/A	Replant canceled due to COVID, ROM 0-40
8	25	M	27.3	12 I&Ds, STSG	PI	MRSE	Extensor mechanism disruption	130	N	N/A	Transitioned care to outside surgeon, ROM 5-55
9	56	M	28.7	Multiple I&Ds	APT	MRSA	Continued swelling, spacer instability	194	N	N/A	Not medically cleared for reimplantation, 10-80
10	46	M	33.1	I&Dx3, gastrocnemius rotational flap after burn/crush injury	APT	MRSA, polymicrobial	wound dehiscence, recurrent infection necessitating repeat spacer	49	Y	VVC, T cone	AKA after replant, doing well with prosthesis
11	63	M	46.8	I&D, synovectomy	APT	MRSA		103	N	VVC	No gait aids, ROM 0-90
12	56	M	40.9	ATS x2	PI	*Pseudomonas aeruginosa*		53	N	MP	officiating high school football, ROM 0-100
13	42	M	32.5	ORIF tibial plateau fracture	PI	Negative	Wound requiring flap coverage and STSG prior to replant	245	N	VVC	Golfing, swimming, ROM 0-125
14	55	M	28.1	I&D x2	PI	MSSA		147	N	VVC	No gait aids, ROM 0-90
15	61	M	26	Biopsy/curettage/grafting/Steinmann pins	PI	MRSE		56	N	VVC, stacked tibial cones	No gait aids, ROM 0-110
16	79	M	21.8	Pus with incision for TKA 6 wk prior-aborted, ATS I&Dx2	PI	Negative		58	N	VVC	Exercises daily, ROM 0-110
17	45	F	36	ACL Recon, ATS	PI	Negative		450	N	N/A	Doing well with spacer, ROM 0-130
18	60	F	31.47	ACL Recon, ATS	APT	Negative		1136	N	N/A	Doing well, developing some stiffness, ROM 0-90, no pain

ACL, anterior cruciate ligament; APT, all-polyethylene tibia; ATS, arthroscopic knee surgery; I&D, incision and drainage; LCL, lateral collateral ligament; MRSA, methicillin-resistant *Staphylococcus aureus*; MRSE, methicillin-resistant *Staphylococcus epidermidis*; MSSA, methicillin-sensitive *Staphylococcus aureus*; MSSE, methicillin-sensitive *Staphylococcus epidermidis*; ORIF, open reduction and internal fixation; PI, polyethylene insert; ROM, range of motion; STSG, split-thickness skin graft; T cone, tibial cone; TKA, total knee arthroplasty; VVC, varus-valgus constraint.

**Table 2:** Patient demographics.

Patient demographics	N = 18
Age	56.1 ± 12.9 years
Sex	
Male	15/18 (83%)
Female	3/18 (17%)
BMI	33.5 ± 7.9
Comorbid DM	8/18 (44%)
Laterality	
Right	9/18 (50%)
Left	9/18 (50%)
Spacer Components	
APT	8/18 (44%)
PI	10/18 (56%)

APT, all-polyethylene tibia; BMI, body mass index; DM, diabetes mellitus; PI, polyethylene insert.

Demographic information collected included age, sex, body mass index, operative extremity, history of prior knee surgery, and comorbid diabetes mellitus. Spacer-related complications were reviewed during the lifetime of the spacer. Soft-tissue cultures were obtained at the time of spacer placement and/or from synovial fluid aspiration during infection workup. Recurrent infection was determined by clinical evaluation or cultures obtained during reimplantation surgery, if applicable. Spacer cost was calculated as the cost of the implants used based on hospital billing sheets to include the femoral and tibial components as well as any adjunctive Steinmann pins or screws implanted. Spacer longevity was defined as the difference between spacer placement and date of reimplantation or spacer placement and last follow-up if the spacer was retained. Reimplantation constructs were evaluated to determine the degree of implant constraint and the need for femoral or tibial cones. Functional outcomes included knee range of motion and preoperative/follow-up KOOS JR scores. Patient-reported outcome scores were unavailable for 6 preoperative and 1 postoperative patient.

**Figure 1:**
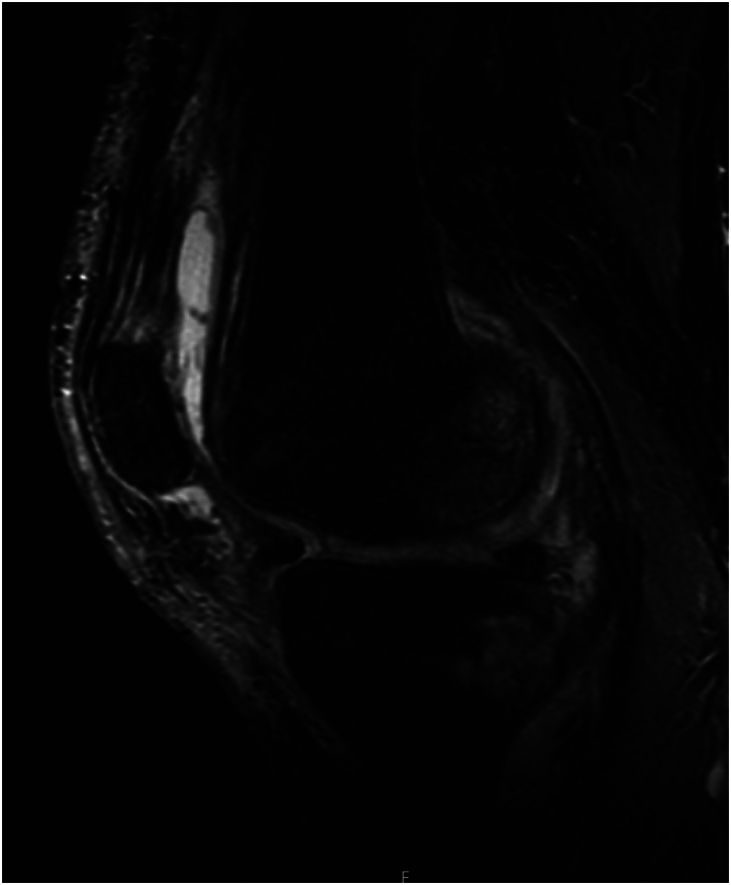
Preoperative MRI showing native posterior tibia and femur osteomyelitis with recurrent effusions, pain, and elevated inflammatory markers. MRI, magnetic resonance imaging.

**Figure 2:**
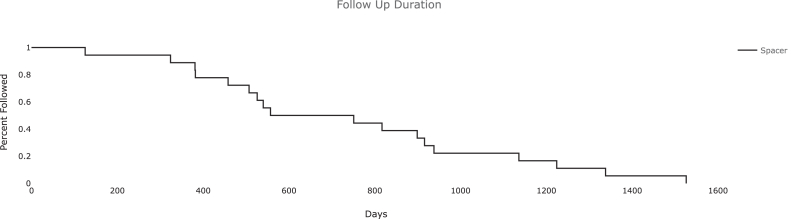
Follow-up duration plotted as a Kaplan Meier survival analysis of all patients that received an articulating spacer.

**Figure 3:**
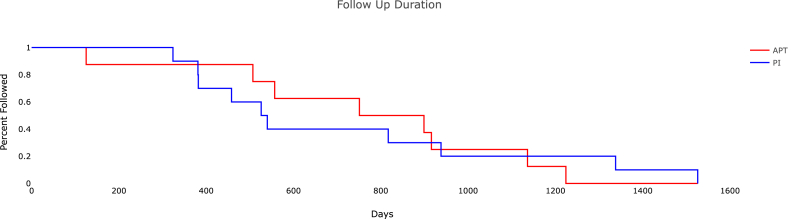
Follow-up duration plotted as a Kaplan Meier survival analysis of APT (all-polyethylene tibia) vs PI (polyethylene insert).

Demographic information (Table 2) for the cohort included an average age of 56.1 (standard deviation 12.9) years, 15/18 male patients, average body mass index 33.5 (standard deviation 7.9), and an equal distribution of right and left knees. Of all patients, 44.4% of them had comorbid diabetes mellitus with 40% (4/10) of the PI subset having diabetes mellitus and 50% (4/8) of the APT subset. Sixteen of the 18 patients had prior open or arthroscopic knee surgery, which included irrigation and debridement (13), trauma (5), and ligament reconstruction (3). One patient developed a septic knee after an intra-articular steroid injection. Using imaging, positive culture laboratory test results, and intraoperative findings, it was determined that all patients had chronically active infections and 6 of these had MRI findings consistent with osteomyelitis (Fig. 1). Average follow-up for all patients in the study was 741 days after spacer placement (Fig. 2). Average follow-up for APT was 764 days and PI was 723 days (Fig. 3).

**Figure 4:**
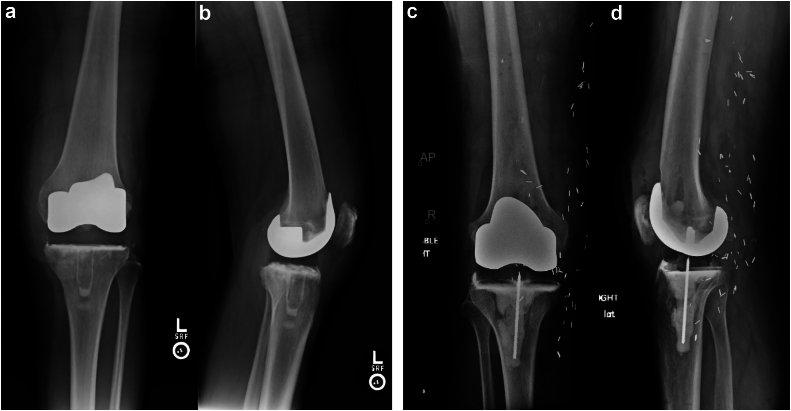
(a and b) Postoperative X-ray showing left all-polyethylene tibia (APT) at 6 weeks. (c and d) Postoperative X-ray showing right polyethylene insert (PI) at 6 weeks.

**Figure 5:**
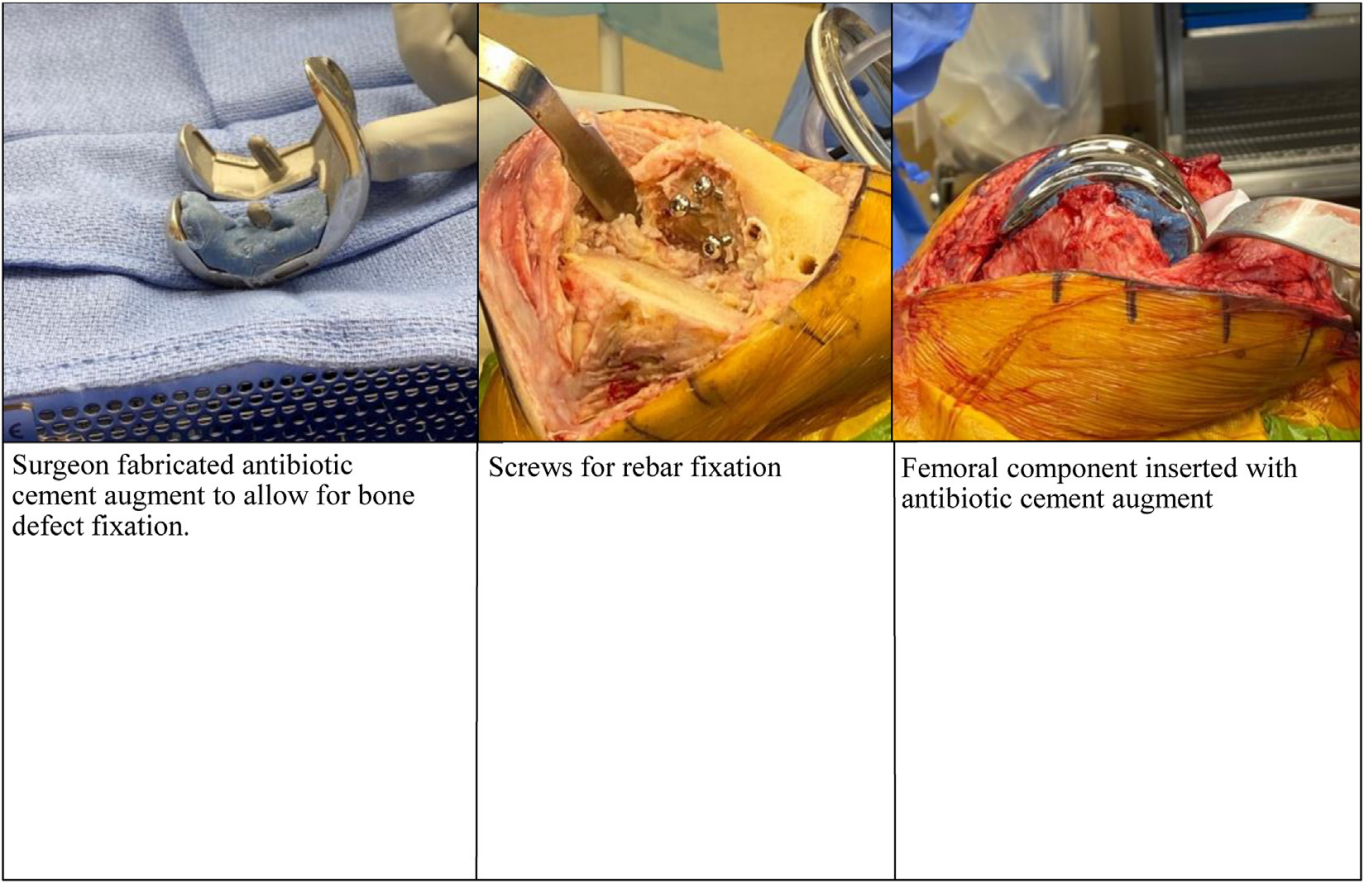
Intraoperative view of surgeon fabricated augment to allow bone defect fixation and Steinmann pin rebaring for proper fit of antibiotic cemented femoral component.

All knees underwent thorough irrigation and debridement of all nonviable or infected bone, removal of previously placed hardware if indicated, and placement of an articulating spacer. The spacer was constructed with a metal femoral component and either an APT or a PI, along with high-dose antibiotic cement (Fig. 4a-d). Adjunctive fixation varied based on the surgeon and the patient findings and consisted of Steinmann pins or screws for rebar fixation, intramedullary cement dowels for antibiotic delivery, and surgeon-fabricated femoral and/or tibial cement augments to address bone defects (Fig. 5). Eight of 18 spacers utilized APT, while 10 of 18 utilized a PI. Six of the 8 APT inserts were the Stryker Triathlon (Stryker, Mahwah, New Jersey), while the remaining 2 were GENESIS II (Smith & Nephew, Watford, England). All APTs were posterior stabilized components (Fig. 4a and b). Six of the 10 PI inserts were the MicroPort Evolution Medial Pivot (MicroPort Orthopedics, Arlington, TN) (Fig. 4c and d), while the remaining 4 were the DJO EMPOWR (Enovis, Wilmington, DE). Of the 8 APT spacers, 2 spacers included antibiotic cemented dowels formed around Steinmann pins and all 10 of the PI spacers included Steinmann pins or screws unitized to the base of a roughened-up PI. Cobalt ® HV bone cement (Enovis, Wilmington, DE) was used with the manual addition of 2-3 grams of vancomycin and 2.4-3.6 grams of tobramycin per 40-gram bag of cement based on surgeon discretion. All patients were treated with culture-directed or broad-spectrum antibiotics as determined by the infectious disease service. Patients were mobilized and allowed to weight bear as tolerated. Patients were evaluated in clinic postoperatively at 2 weeks and again at 6-8 weeks after spacer placement to monitor infection control and candidacy for second-stage reimplantation. If the knee was functioning well, did not have radiographic signs of loosening and infection was controlled, shared decision-making was utilized to determine retention or further surgery based on clinical follow-up at regular intervals.

Of the 12 subjects who went on to revision surgery, constructs consisted of 9 varus-valgus constrained (5 Exprt Revision, Enovis, Wilmington, DE and 4 Truliant Revision total knee systems, Exactech, Gainesville, FL), 2 hinged knee prostheses (MicroPort Guardian Revision TKA system, Arlington, TN), and 1 medial pivot revision knee (MicroPort Evolution TKA system, Memphis, TN). Cemented stems were used in all cases for both the femoral and tibial components.

**Figure 6:**
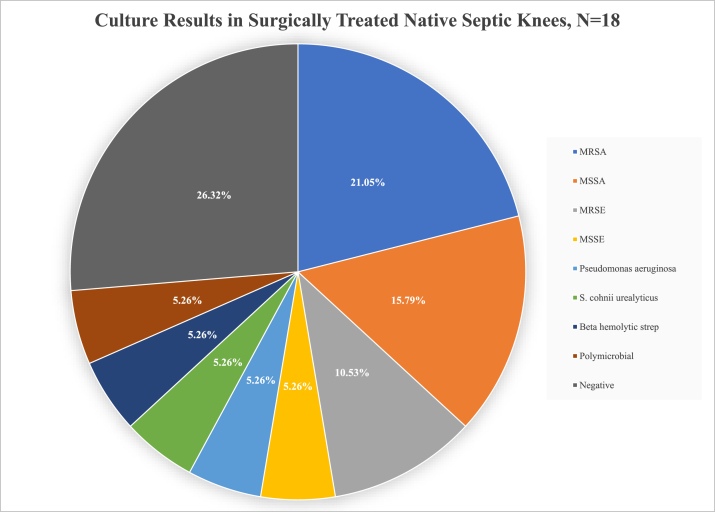
Culture results in surgically treated native septic knees, N = 18.

The most common infecting organism identified at the time of spacer placement was methicillin-resistant *Staphylococcus aureus* (MRSA) (4), followed by methicillin-sensitive *Staphylococcus aureus* (MSSA) (3), and methicillin-resistant *Staphylococcus epidermidis* (2). There were individual cases of methicillin-sensitive staphylococcus epidermidis, *Pseudomonas aeruginosa*, beta-hemolytic *Streptococcus*, *Staphylococcus cohnii urealyticus*, and a polymicrobial infection (Fig. 6). Five chronically infected knees yielded negative cultures at the time of spacer placement (Table 1).

## Results

Six of 18 (33%) patients have retained their spacers with an average spacer duration of 425 days (3 APT, 3 PI). The average spacer survivorship duration is 629 days for the APT group and 222.7 days for the PI group (*P* = .265). The retained spacer group had an overall lower rate of culture positive samples, osteomyelitis, and diabetes; however, the **(Δ)** KOOS JR was similar to that of the reimplantation group (Table 3). Three of the 6 patients that retained their spacers did so due to satisfaction with results and positive outcome. Two patients transitioned to outside care, and 1 could not have reimplantation surgery due to untreated coronary artery disease and never came back for follow-up in the year after surgery. Five APT patients (62.5%) and 7 PI patients (70%) underwent reimplantation as part of the 2-stage treatment of chronic, septic knee arthritis (12 total). The average time that both spacers remained for knees that underwent reimplantation was 106 days. The mean time before reimplantation for APT was 94.8 days and for PI was 114.6 days (*P* = .588). The maximum duration before reimplantation was 174 days for the APT group and 245 days for the PI group.

**Table 3:** Summary of survivorship.

Treatment								
Treatment received	Total (18/18)	Culture (+)	Culture (−)	Osteomyelitis	Diabetes	Average preoperative KOOS JR	Average follow-up KOOS JR	Average change (Δ) in KOOS JR
Maintained articulating spacer	33% (6/18)	66% (4/6)	33% (2/6)	17% (1/6)	25% (2/8)	42.2	59.6	17.5
Reimplantation	66% (12/18)	83% (10/12)	17% (2/12)	83% (5/6)	75% (6/8)	48.6	64.9	16.3

N = 18.

**Table 4:** Spacer KOOS JR scores.

Spacer type			
Spacer	Average pre-operative KOOS JR	Average follow-up KOOS JR	Average change (Δ) in KOOS JR
APT	53.6	51.2	−2.4
PI	41.3	71.3	30.0

N = 18.

**Table 5:** Summary of articulating spacer.

Spacer type										
Spacer	Reimplantation total (12/18)	Varus valgus	Hinge	Medial pivot	T cones used	Culture (+)	Culture (−)	Average pre-operative KOOS Jr	Average follow-up KOOS Jr	Average change (Δ) in KOOS Jr
APT	62.5% (5/8)	100% (5)	0	0	60% (3)	100% (5/5)	0	52.4	50.9	−1.5
PI	70% (7/10)	57% (4)	28% (2)	14% (1)	42% (3)	71% (5/7)	29% (2/7)	43.6	72.9	29.3

N = 18.

Five of 18 (28%) patients experienced complications related to the spacer; 3 in the PI group and 2 in the APT group. These complications included delayed wound healing requiring surgical intervention (2 PI), arthrofibrosis (1 PI), recurrent infection with repeat debridement and spacer exchange (1 APT), extensor mechanism disruption (1 PI), and spacer instability (1 APT). Multiple of these complications occurred in 1 patient who received an APT with preoperative cultures positive for MRSA and polymicrobial organisms. Later, this patient was found to have wound dehiscence leading to infection requiring spacer replacement and eventually AKA. Besides this event, the other 17 patients (87.5% of APT and 100% of PI) did not experience infection recurrence at an average follow-up of 2 years. Of the patients who experienced spacer-related complications, 3 grew MRSA, 1 methicillin-resistant *Staphylococcus epidermidis*, and 1 was culture-negative in preoperative or intraoperative samples taken.

**Figure 7:**
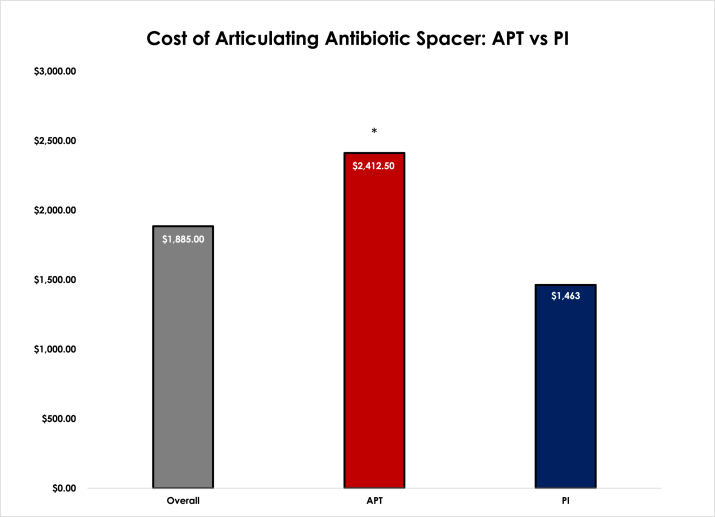
Cost of Articulating Antibiotic Spacers: APT (all-polyethylene tibia) vs PI (polyethylene insert). (∗) Signifies a statically significant difference.

The average cost of all antibiotic spacers in the study was $1885.00. The cost was significantly greater for APT spacers at $2412.50 compared to PI spacers at $1463.00. (*P* < .001) (Fig. 7). Two of the 8 APT spacers were constructed using additional Steinmann pins into the base of the keel, while all 10 of the PI spacers utilized a screw or Steinmann pin inserted into the undersurface of the polyethylene insert (4 with a screw, 6 with a Steinmann pin) for added stability.

KOOS JR scores at the most recent follow-up for 17 of 18 patients (excluding the AKA) receiving a spacer for native joint infection averaged 62.7. The mean change in KOOS JR scores from preoperative to most recent follow-up was 17.3, which is greater than the median minimum clinically important difference (MCID) for KOOS JR scores for joint replacement [17]. When comparing KOOS JR of APT vs PI, Table 4 showed PI performed over 30 points higher in most recent follow-up. When comparing patient who kept their spacer vs those who went on to second-stage reimplantation, Table 3 showed only a 1-point difference in favor of patients who retained their spacer. Range of motion (ROM) after either sustained spacer or reimplantation was attained at most recent follow-up with an average of 101.2 ± 24.2 degrees (Table 1). The ROM for patients that received APT was 104.3 ± 16.6 and ROM for patients that received PI was 99.0 ± 28.2 (Table 1).

## Discussion

Treating patients with septic arthritis in native knees can pose many challenges. Established techniques employed for the treatment of periprosthetic joint infection may be useful in the treatment of this difficult problem, such as 2-stage exchange protocols with various types of antibiotic spacers, and long-term antibiotics [[1], [2], [3], [4], [5], [6], [7], [8], [9], [10], [11], [12], [13]]. We do not have a strict institutional approach to this problem as there are many patient-specific factors considered for these cases. Our surgeons have overall moved more toward the use of PI for spacers especially since this allows for a larger liner size (most APT liners have a maximum thickness of 15 mm). One approach taken for our patients with native septic arthritis is fabricating an adequate cement job with our constructs and appropriately balancing the flexion and extension spaces with the intent to allow patients to keep them as a 1.5 functional spacer moving forward. In general, the decision for TKA is based on the presence of underlying arthritic changes and supplemental findings, such as the presence of osteomyelitis or evidence of bone loss. Overall, 3 of our surgeons leave spacers in if not symptomatic and functioning well, while 1 surgeon prefers the traditional two-stage procedure. This is a similar approach utilized Hooper et al [10]. That combination of factors, plus the duration of symptoms, combine to drive the shared decision-making for primary articulating spacer.

Patients with a history of native knee septic arthritis have a higher incidence of PJI after primary TKA [18], so it may be beneficial to opt for a 2-stage approach to attempt to eradicate the infection. A study by Bettencourt et al. [19] found a 6.1-fold increase in risk of PJI for patients undergoing TKA with a history of native knee septic arthritis. Our study demonstrates a 94.4% (17/18) success rate of 2-stage and 1.5-stage [14] exchange arthroplasty in the setting of chronically infected native knees for patients who received a functional MoP articulating antibiotic spacer utilizing 2 different types of constructs. Most patients (14/18) remained free from infectious sequelae, revision, or failure at 2 years (3 maintained functional spacers, 11 went through with knee reimplantation). Functionally, all 14 patients who were successfully treated in this fashion exhibit full active extension and an average flexion of 110 ± 13.8 degrees. This is similar to previously published results by Kunze et al. [5] who reported 100.5 ± 17.1 degrees of knee range of motion postoperatively in 30 cement-molded and static knee spacers. We noted 5 patients who experienced spacer-related complications, including wound issues requiring reoperation (2), arthrofibrosis (1), recurrent infection with repeat debridement and spacer exchange (1), extensor mechanism disruption (1), and spacer instability (1). This mirrors the complication profile reported by Kunze et al [5] which also included spacer instability/dislocation, recurrent infection with repeat spacer, and arthrofibrosis requiring manipulation under anesthesia.

According to the KOOS JR PASS (patient acceptable symptom state) criteria reported by Kunze et al [20] for primary knee arthroplasty, the threshold of 2-year follow-up scoring is 63.7. The PASS criteria were achieved by the reimplantation PI spacer group at 72.9 (Table 5), the overall reimplantation group at 64.9 (Table 3), and the overall PI spacer group at 71.3 (Table 4). The largest KOOS JR score changes in preoperative to postoperative follow-ups were achieved by both the overall PI and reimplantation PI groups at **Δ**30.0 and **Δ**29.3, respectively (Table 3, Table 4). According to Hung et al [17], the median MCID for change (Δ) in KOOS JR is said to be 15.1 which was achieved by the reimplantation PI spacer group (29.3), the overall reimplantation group (16.3), the maintained articulating spacer group (17.5), and the overall PI spacer group (30.0) (Table 3, Table 4, Table 5). Interestingly, while the maintained articulating spacer group did not achieve PASS criterion, they did achieve MCID and had a greater change **(Δ)** in KOOS JR follow-up compared to the reimplantation group (Table 3). The maintained articulating spacer group had a lower baseline KOOS JR and achieved a greater change possibly limiting the chance to achieve PASS criteria.

In the group that underwent reimplantation, 33% had osteomyelitis and 83% had a culture-positive sample at the initial surgery for articulating spacer placement compared to the maintained articulating spacer group in which 16% had osteomyelitis and only 66% were culture positive. The majority (83%) of patients with osteomyelitis underwent reimplantation (Table 3). The reason as to why the APT group had an average KOOS JR change **(Δ)** of −2.4 may be due to reimplantation constraint, culture rate, or baseline function. All APTs were reimplanted with varus-valgus constraining constructs, while the PI group only had 57% reimplanted with this level of constraint (Table 5). The APT group also had a 100% culture positive rate, while only 71% of the PI group were culture positive. Osteomyelitis rates were similar between the 2 groups (25% APT, 40% PI). It is possible that the infection profile or degree of bone loss could contribute to these subtle differences.

Twelve of 18 patients underwent second-stage reimplantation, while 6 patients retained their spacers at 2 years average follow-up. Hernandez et al [14] evaluated a series of 31 articulating spacers placed for PJI with the intent for long-term spacer retention and they found 81% of spacers remained in situ at 2.7 years. A recent small series by Hooper et al. [10] reported that 5 out of 6 MoP spacers placed for native knee infection were retained at 3 years. Our retention rate may differ from this due to surgeon intent for planned second-stage reimplantation or simply due to the small sample size. The 6 patients that retained their spacer had lower comorbidities including culture-positive samples, osteomyelitis, and diabetes (Table 3). Interestingly the (Δ) in KOOS JR of retained spacers vs reimplantation was very similar at 17.5 and 16.3, respectively. Three of the patients that retained their spacers did so due to satisfactory results in outcome included an average ROM of 0 to 110 ± 16.3. The other 3 patients retained their spacers due to either transfer of care or untreated coronary artery disease and lack of follow-up. Our previous study by Kinder et al. showed no difference in complications and lower costs using the PI rather than the APT for PJI. For that reason, our surgeons have overall moved more toward the use of PI for primary knee spacers. PIs also allow for a thicker liner size when necessary to make up space for bone loss. At the time of our study, there did not seem to be a clear reason and much of what went into the spacer usage was surgeon preference. Notably, we noticed that survivorship was related primarily to patient function and happiness with the spacer and no desire for further surgery.

All but 1 of the patients in our study (17/18) were without infection recurrence by culture or clinical evidence at 2 years of follow-up, which aligns with the findings from Hooper et al [10], showing 15/16 (94%) articulating and static spacer patients remained infection-free at an average of 3-year follow-up. Our results further support the use of an articulating antibiotic spacer, with either an APT or PI construct, for the treatment of chronic native knee infection.

The one instance of recurrent infection is in a complex patient with obesity, and type 2 diabetes mellitus. The patient initially sustained third degree burns and crush injuries from the lower extremity to the gluteal area that required multiple surgeries and soft-tissue coverage during the initial admission from the injury. Over the course of 1 month, this patient received 9 total surgeries including fasciotomies, debridement, arthrotomies, and a gastrocnemius flap surgery. Subsequently, an articulating spacer was placed 2 months after discharge from the initial hospital stay. The patient had a recurrent infection requiring articulating antibiotic spacer exchange with APT 49 days later, the shortest of all spacer durations in our cohort. Indications for this revision were chronic infected knee and great risk of complications given past medical history of burn and crush injuries. The patient returned for the second-stage reimplantation with a varus-valgus constraint TKA 3 months later. The patient then underwent repeat debridement, irrigation, and revision spacer placement 6 months later due to persistent drainage and failure of conservative treatment measures. One year after the initial traumatic crush wounds, the patient ultimately underwent an above-knee amputation due to recurrent polymicrobial chronic knee infection. The severity of infectious, systemic, and local extremity trauma placed this patient at a grade III-B-3 according to the McPherson classification, which has been associated with very poor infection clearance rates [21].

The initial pre-operative or intraoperative culture negative rate was 28% (5/18), which is similar to 26% reported by Kunze et al [5], necessitating the use of empiric antibiotic treatment. We found an overall predilection for staphylococcal species (MRSA, MSSA) in our series. MRSA was the most common infectious organism consisting of 22.2% (4/18) of all identified species, while MSSA was the second most common at 16.7% (3/18). These infection profiles are in line with the 23.3% MRSA and 11.1% MSSA reported by Jaffe et al. [22] looking at culture results in (N = 80) surgically treated native septic knees. About 53.8% (7/13) of positive cultures contained *Staphylococcus aureus* which is the most common cause of native knee septic arthritis [13]. About 60% (3/5) of the patients with complications had an MRSA infection. MRSA has been shown to cause more damage, have higher toxicity, and is associated with more complications and higher mortality than MSSA [23]. Although the sample size is small in our cohort, this may shine light onto the correlation between MRSA infections in the native knee and higher-complication risks during articulating spacer surgeries.

Our study has several limitations. This is a retrospective study comprised a small patient population due to the low prevalence of chronic native septic arthritis. Despite this, it is currently the largest series to be reported utilizing modern MoP articulating spacers to manage chronic native knee infections. Multicenter prospective studies are needed to improve the evidence for utilizing this treatment protocol as the data on this topic are currently quite limited. Surgeon intent for long-term spacer retention vs planned reimplantation may also skew the results regarding spacer retention. Additionally, patients’ lack of willingness to undergo a second-stage reimplantation may have been affected by the coronavirus disease 2019 pandemic, leading to increased retention rates of functional spacers. Longer clinical and radiographic follow-up will further elucidate the long-term success rate of this treatment protocol.

## Conclusions

Our results demonstrate that utilizing a 2-stage protocol similar to the treatment of PJI in TKA is a viable option for patients with chronic septic knee arthritis. The PI implant performed better with greater differences in overall KOOS JR, MCID, PASS criteria and is significantly cheaper than the APT implant. Both maintained articulating spacer and 2-stage reimplantation groups achieved KOOS JR scores above the MCID, proving spacer retention is an option in well-functioning spacers. More research is needed to further optimize treatment for this difficult clinical challenge.

## Funding

Research reported in this publication was supported by the 10.13039/100000057National Institute of General Medical Sciences of the 10.13039/100000002National Institutes of Health under Award Number P20GM125503. The content is solely the responsibility of the authors and does not necessarily represent the official views of the National Institutes of Health. In addition, the Bill and Betty Petty Research Fund at the 10.13039/100008519University of Arkansas for Medical Sciences supported this work.

## Conflicts of interest

C. L. Barnes receives royalties from DJO and Zimmer; is a MicroPort Orthopaedics paid consultant; receives stock or stock options from Avant-garde Health; BEKHealth; Clozex Medical; Excelerate Health Ventures; Green OR; Hayle Surgical; In2Bones SAS; MiCare Path; Plakous; Ride Health; ROM3 Rehab, LLC; Sleep Partners, LLC; Sniffle; serves in the J*ournal of Knee Surgery*; *Journal of Surgical Orthopaedic Advances* Medical/Orthopaedic publications editorial/governing board; is a American Association of Hip and Knee Surgeons, HipKnee Arkansas Foundation, and Southern Orthopaedic Association board member; B. M. Stronach receives royalties from MiCare Path, Sawbones/Pacific Research Laboratories, and Tightline Development LLC; serves in the DJ Orthopaedics speakers bureau; is a DJ Orthopaedics and Johnson & Johnson paid consultant; receives stock or stock options from Joint Development LLC; serves in the JBJS-Br Medical/Orthopaedic publications editorial/governing board; is a AAOS, American Association of Hip and Knee Surgeons board member; S. C. Mears receives stock or stock options from Delta Ortho LLC; serves in the *Journal of the American Geriatrics Society*, SAGE Medical/Orthopaedic publications editorial/governing board; is a Fragility Fracture Network board member; J. B. Stambough receives royalties from Signature Orthopaedics, is a Smith & Nephew paid consultant, receives other financial or material support from Smith & Nephew, serves in the *Journal of Arthroplasty* editorial board, is a American Association of Hip & Knee Surgeons (AAHKS) Education Committee board member.

For full disclosure statements refer to https://doi.org/10.1016/j.artd.2024.101329.

## CRediT authorship contribution statement

**Levent A. Ozdemir:** Data curation, Formal analysis, Writing – original draft, Writing – review & editing. **Andrew E. Apple:** Data curation, Formal analysis, Writing – original draft. **C. Lowry Barnes:** Conceptualization, Data curation, Formal analysis, Investigation, Methodology, Project administration, Validation, Writing – original draft, Writing – review & editing. **Benjamin Stronach:** Conceptualization, Data curation, Formal analysis, Investigation, Methodology, Project administration, Supervision, Validation, Writing – original draft, Writing – review & editing. **Simon C. Mears:** Conceptualization, Data curation, Formal analysis, Investigation, Methodology, Project administration, Supervision, Validation, Writing – original draft, Writing – review & editing. **Jeffrey B. Stambough:** Conceptualization, Data curation, Formal analysis, Investigation, Methodology, Project administration, Resources, Supervision, Validation, Writing – original draft, Writing – review & editing.

## Appendix A Supplementary data

Conflict of Interest Statement for Barnes

Conflict of Interest Statement for Stronach

Conflict of Interest Statement for Apple

Conflict of Interest Statement for Ozdemir

Conflict of Interest Statement for Stambough

Conflict of Interest Statement for Mears
