# Supplementary material for: Functional Articulating Antibiotic Spacers for Chronic Native Septic Knee Arthritis
Source: Arthroplast Today. 2024 Jun 27;27:101329. doi: 10.1016/j.artd.2024.101329 (PMC11282414; doi:10.1016/j.artd.2024.101329)
Supplement: Conflict of Interest Statement for Stronach [file mmc2.docx]

# CONFLICT OF INTEREST STATEMENT

***American Association of Hip and Knee Surgeons***

(Adopted from the American Academy of Orthopaedic Surgeons disclosure statement)

The following form **must be filled out completely and submitted by each author (example, 6 authors, 6 forms).**

**All items require a response. If there is no relevant disclosure for a given item, enter "*None*.”**

Manuscript Title: Functional Articulating Antibiotic Spacers for Chronic Native Septic Knee Arthritis

1. Royalties from a company or supplier (The following conflicts were disclosed)

MiCare Path, Sawbones/Pacific Research Laboratories, Tightline Development LLC

2. Speakers bureau/paid presentations for a company or supplier (The following conflicts were disclosed)

DJ Orthopaedics

3A. Paid employee for a company or supplier (The following conflicts were disclosed)

None

3B. Paid consultant for a company or supplier (The following conflicts were disclosed)

DJ Orthopaedics, Johnson & Johnson

3C. Unpaid consultants for a company or supplier (The following conflicts were disclosed)

None

4. Stock or stock options in a company or supplier (The following conflicts were disclosed)

Joint Development LLC

5. Research support from a company or supplier as a Principal Investigator (The following conflicts were disclosed)

None

6. Other financial or material support from a company or supplier (The following conflicts were disclosed)

None

7. Royalties, financial or material support from publishers (The following conflicts were disclosed)

None

8. Medical/Orthopaedic publications editorial/governing board (The following conflicts were disclosed)

JBJS-Br

9. Board member/committee appointments for a society (The following conflicts were disclosed)

AAOS, American Association of Hip and Knee Surgeons

**Each author must sign AND print or type his/her name, date and submit a separate form**

Benjamin M. Stronach, MD Benjamin M. Stronach, MD August 21, 2023

Author Name (Print or Type) Author Signature Date
