# Supplementary material for: Functional Articulating Antibiotic Spacers for Chronic Native Septic Knee Arthritis
Source: Arthroplast Today. 2024 Jun 27;27:101329. doi: 10.1016/j.artd.2024.101329 (PMC11282414; doi:10.1016/j.artd.2024.101329)
Supplement: Conflict of Interest Statement for Ozdemir [file mmc4.docx]

# INDIVIDUAL CONFLICT OF INTEREST STATEMENT

(Adopted from the American Academy of Orthopaedic Surgeons disclosure statement)

The following form **must be filled out completely and submitted by each author (example, 6 authors, 6 forms).**

**All items require a response. If there is no relevant disclosure for a given item, enter "*None*.”**

**Manuscript Title:** Functional Articulating Antibiotic Spacers for Chronic Native Septic Knee Arthritis

1. Royalties from a company or supplier (The following conflicts were disclosed)none

2. Speakers bureau/paid presentations for a company or supplier (The following conflicts were disclosed)none

3A. Paid employee for a company or supplier (The following conflicts were disclosed)none

3B. Paid consultant for a company or supplier (The following conflicts were disclosed)none

3C. Unpaid consultants for a company or supplier (The following conflicts were disclosed)none

4. Stock or stock options in a company or supplier (The following conflicts were disclosed)none

5. Research support from a company or supplier as a Principal Investigator (The following conflicts were disclosed)none

6. Other financial or material support from a company or supplier (The following conflicts were disclosed)none

7. Royalties, financial or material support from publishers (The following conflicts were disclosed)none

8. Medical/Orthopaedic publications editorial/governing board (The following conflicts were disclosed)none

9. Board member/committee appointments for a society (The following conflicts were disclosed)none

**Each author must sign AND print or type his/her name, date and submit a separate form**

Levent A. Ozdemir Levent A. Ozdemir August 21, 2023

Author Name (Print or Type) Author Signature Date
